# Circulating lipocalin-2 across the adult lifespan

**DOI:** 10.1093/jbmrpl/ziae162

**Published:** 2024-12-10

**Authors:** Carlie Bauer, Cassandra Smith, Sara Vogrin, Andrew S Palmer, Mary Woessner, Shanie Landen, Macsue Jacques, Elizabeth Byrnes, Nir Eynon, Marc Sim, Joshua R Lewis, Itamar Levinger

**Affiliations:** Institute for Health and Sport, Victoria University, Melbourne, VIC 3011, Australia; Australian Institute for Musculoskeletal Science, Victoria University, University of Melbourne, St Albans, VIC 3021, Australia; Nutrition & Health Innovation Research Institute, Edith Cowan University, Joondalup, WA 6027, Australia; Medical School, The University of Western Australia, Perth, WA 6009, Australia; Australian Institute for Musculoskeletal Science, Victoria University, University of Melbourne, St Albans, VIC 3021, Australia; Department of Medicine, Western Health, The University of Melbourne, St Albans, VIC 3021, Australia; Department of Medicine, St Vincent’s Hospital Melbourne, The University of Melbourne, Fitzroy, VIC 3065, Australia; Institute for Health and Sport, Victoria University, Melbourne, VIC 3011, Australia; Institute for Health and Sport, Victoria University, Melbourne, VIC 3011, Australia; Centre for Endocrinology and Metabolism, Hudson Institute of Medical Research, Melbourne, VIC 3168, Australia; Institute for Health and Sport, Victoria University, Melbourne, VIC 3011, Australia; Australian Regenerative Medicine Institute, Monash University, Clayton, VIC 3168, Australia; PathWest, QEII Medical Centre, Perth, WA 6009, Australia; Australian Regenerative Medicine Institute, Monash University, Clayton, VIC 3168, Australia; Nutrition & Health Innovation Research Institute, Edith Cowan University, Joondalup, WA 6027, Australia; Medical School, The University of Western Australia, Perth, WA 6009, Australia; Nutrition & Health Innovation Research Institute, Edith Cowan University, Joondalup, WA 6027, Australia; Medical School, The University of Western Australia, Perth, WA 6009, Australia; Centre for Kidney Research, Children’s Hospital at Westmead School of Public Health, Sydney Medical School, The University of Sydney, Sydney, NSW 2006, Australia; Institute for Health and Sport, Victoria University, Melbourne, VIC 3011, Australia; Australian Institute for Musculoskeletal Science, Victoria University, University of Melbourne, St Albans, VIC 3021, Australia

**Keywords:** aging, clinical trials, bone interactors, endocrine, cytokines

## Abstract

Lipocalin-2 (LCN2), a hormone produced by adipocytes, osteoblasts, and renal tubular cells, is implicated in age-related diseases, including cardio-metabolic disease. To understand the role LCN2 may play in pathological states, we first need to elucidate the relationship between circulating LCN2 with indices of cardio-metabolic health during “normal” aging. This study examined the relationship between serum levels of LCN2, age, and cardio-metabolic measures across the adult lifespan in males and females. We conducted a pooled cohort analysis including 124 community-dwelling males (*n* = 52) and females (*n* = 72) (age 20–87 yr, median BMI 25.92 [23.04, 29.81] kg/m^2^). Serum LCN2 was analyzed using a two-step chemiluminescent microparticle monoclonal immunoassay. The relationship between LCN2 and age was evaluated by linear regression and cubic spline. Simple linear regressions were performed to investigate the relationship between LCN2 and the following variables: BMI, VO_2peak_, serum glucose, and body composition (DXA). For every 1 yr increase in age, LCN2 levels were 0.26 mg/L higher (*P* = .007, 95% CI [0.07, 0.45]). Each 1 unit increase in BMI (kg/m^2^) was associated with 0.88 mg/L higher LCN2 levels (*P* = .027, [0.10, 1.66]) and each 1 unit increase in VO_2peak_ (mL/kg/min) was associated with 0.38 mg/L lower LCN2 (*p* = .003, [−0.63, −0.13]).There was no significant relationship between LCN2 and sex, glucose levels or body composition (all *p* > .05). LCN2 increased linearly across the adult lifespan while it decreased as fitness level increased. Future research should build on these findings to determine whether LCN2 can be used as a biomarker for chronic disease and if exercise can mitigate age-related disease associated with LCN2 changes.

## Introduction

The World Health Organization reported a 6-year increase in life expectancy (lifespan) between 2000 and 2019.^1^ However, increases in healthy life expectancy (healthspan), which is years lived in full health, has not increased to the same degree.^1^ Aging is associated with the deterioration of physiological processes contributing to chronic disease, such as cardio-metabolic disease, osteoporosis, and sarcopenia,^2^^,^^3^ with age-related diseases accounting for 51.3% of all global disease burden.^4^

Bone-muscle-fat interaction is implicated in age-related metabolic disease.^5^^,^^6^ There is accumulating evidence that bone, as an endocrine organ, plays a role in cardio-metabolic function.^7^ While the specific pathways and hormones involved in these interactions are largely unknown, evidence suggests that lipocalin-2 (LCN2) is involved.^7^ Although LCN2 is largely considered an adipokine produced by adipocytes, it is also produced and released into circulation by multiple cell types, such as bone forming cells (osteoblasts) and renal tubular cells. It is hypothesized that under “normal” physiological conditions LCN2 is predominantly produced by bone, whereas in pathological states such as obesity and type 2 diabetes, production from adipose tissue is increased.^7^^,^^8^ Circulating LCN2 is also upregulated after kidney injury and is considered a biomarker for acute kidney injury and chronic kidney disease severity.^9^^,^^10^ A growing body of evidence suggests circulating LCN2 levels are increased in pathological cardio-metabolic states, independent of its role in kidney injury.^11^^,^^12^

Despite the reported relationship of LCN2 with cardio-metabolic disease and acute kidney injury, to date it is not clear whether circulating levels of LCN2 change during normal aging across the adult lifespan. We previously reported a “U” shaped relationship with changes across the adult lifespan in other bone related peptides, osteoglycin, and osteocalcin, which are implicated in cardio-metabolic disease.^13^^,^^14^ Given that many cell types that release LCN2, it is unclear whether a similar pattern will be observed. Previous studies have suggested that higher circulating LCN2 levels are associated with increased age in highly fit males who are ultra-athletes (17-54 yr), healthy women (50-85 yr),^15^ and community-dwelling older women (>70 yr).^16^ However, no study has examined differences in LCN2 levels over the entire adult lifespan accounting for sex and increases in age.

There is emerging evidence that LCN2 is a mechanoresponsive protein, with evidence of a relationship between increased serum LCN2 and mechanical unloading during bed-rest.^17^ Epidemiological studies have reported that lower physical activity levels exhibit increased circulating LCN2 in older, community-dwelling women.^18^ On the other hand, some reported that LCN2 remains stable in response to moderate intensity exercise training in middle-aged overweight and obese men and women.^19^ Therefore, we seek to clarify whether LCN2 levels and aerobic capacity (VO_2peak_) are related across the adult lifespan.

The aim of the current study was to investigate the differences of circulating LCN2 levels across the adult lifespan and secondarily, to examine the relationship between LCN2 and BMI, VO_2peak_, glucose, sex, and body composition.

## Materials and methods

This study is a cross-sectional pooled analysis. Data from 124 community-dwelling adult males (*n* = 52) and females (*n* = 72) aged 20-87 yr who were free of major disease and not participating in vigorous or high intensity exercise were included from the following studies: The Gene Smart study (aged 18-45 yr, *n* = 44),^20^^,^^21^ the Wellderly study (aged 60 yr or older, *n* = 37),^22^ the Wellderly study 2 (aged 45 yr or older, *n* = 33) and a smaller exercise study with middle-aged males (40-70 yr, *n* = 10) conducted at Victoria University, Melbourne.^23^ The Wellderly study 2 had similar protocols to the initial Wellderly study with the addition of exclusion for current smokers or those who quit in the past 12 months. Two participants from the Wellderly study (aged 60 yr or older) were current smokers. All participants completed informed consent. Ethics was approved by the Victoria University ethics committee for all studies and three of the studies were clinical trials registered with the Australia New Zealand Clinical Trials Registry: ACTRN12618001756213, ACTRN12622000337774, and ACTRN12613000706774. Trials were conducted in accordance with the ethical standards of the Helsinki Declaration.

Participants attended the laboratory in the morning following an overnight fast and were asked to avoid exercise for at least 48 hr prior to attending.

### Biochemistry analysis

Fasting blood samples were collected in serum separator tubes, clotted for 10 min, then separated into serum by centrifugation (10 min at 3500 rpm, 4 °C). Serum was aliquoted and immediately stored at −80 °C until analysis. Serum LCN2 was analyzed using a two-step chemiluminescent microparticle monoclonal immunoassay on an automated platform (Abbott Diagnostics) in 11 runs over a 3-wk period. Inter-assay CV’s were 5.47% (20 ng/L), 2.81% (190 ng/L), and 2.86% (1170 ng/L). Serum glucose was measured using an automated analyzer (YSI 2300 STAT Plus Glucose & Lactate Analyzer).

### Graded exercise test

All studies included a graded exercise test with gas analysis via cycle ergometer to obtain VO_2peak_, a gold standard measure of aerobic capacity. Graded exercise test protocols for each study were previously described.^20–23^

### Dual-energy X-ray absorptiometry

Total body composition (fat mass, lean mass, and body fat %) was assessed using DXA in a subset of participants (*n* = 70): Wellderly (DXA, Hologic, Horizon A, software version 5.6.0.4) and Wellderly 2 cohorts (GE Lunar iDXA, GE Healthcare). DXA data were unavailable for the other study cohorts with participants aged 45 yr or younger.

### Statistical analysis

Statistical analysis was completed using Stata 17. Participant characteristics are expressed as median (IQR) for continuous variables or % for frequencies. Relationship between LCN2 and age was evaluated by linear regression and cubic splines using 1-4 knots. Models were compared by comparing deviance using a likelihood ratio test, as well as comparison of Akaike and Bayesian information criteria (AIC and BIC, respectively, Table 2).

Scatter plots were visually assessed for linearity for other variables. Simple linear regressions were analyzed to investigate the relationship between LCN2 levels and other variables (BMI, VO_2peak_, serum glucose, and DXA results). Reference ranges for serum LCN2 were derived from analysis with age as a continuous variable, using 95% prediction interval from linear regression. To ease the interpretation, age was divided into 10-yr age groups and the reference range for the mean age within age group was presented. For all statistical analyses, *p* values < .05 were considered statistically significant.

## Results

This study included 124 participants (male *n* = 52 [41.9%], female *n* = 72 [58.1%]; median age 57 [37, 70] yr; median BMI 25.92 [23.04, 29.81] kg/m^2^). There was a significant difference in the mean age between male and female participants (*p* = .039). Participant characteristics are presented in Table 1.

**Table 1:** Participant characteristics (*n* = 124).

Variable	
**Sex (*n* (%))**	Male 52 (41.9) Female 72 (58.1)
**Age (yr)**	57 (37, 70)
**BMI (kg/m^2^)**	25.92 (23.04, 29.81)
**VO_2peak_**	24.95 (18.8, 40.74)^a^
**Glucose (mmol/L)**	5.51 (5.27, 6.04)^b^
**LCN2 (mg/L)**	67.4 (57.25, 79)
Total body fat (%)^*^	38.15 (34.3, 45.1)^c^
Total body fat mass (kg)^*^	28.13 (22.24, 34.55)^c^
Total body lean mass (kg)^*^	41.57 (37.45, 48.73)^c^

Data are presented as median (IQR) for continuous variables or *n* (%) for frequencies.

Abbreviation: LCN2, lipocalin-2.

a *n* = 120.

b *n* = 103.

c *n* = 70.

**Figure 1:**
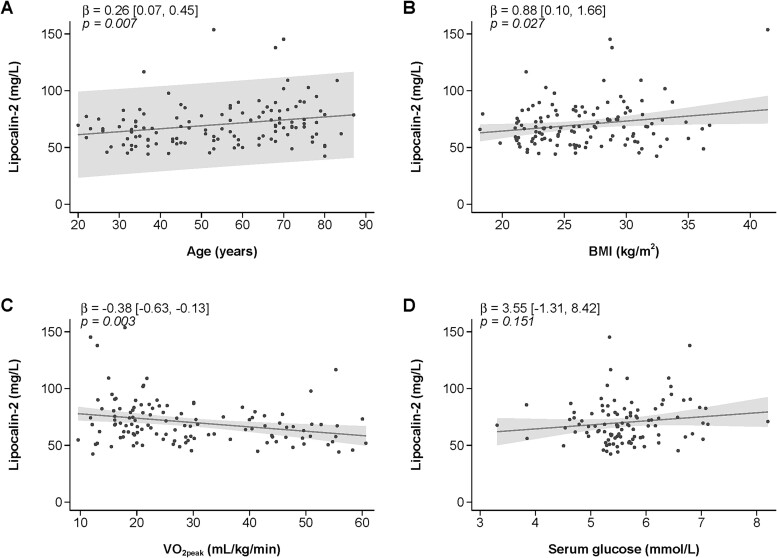
Linear regression of the relationship between lipocalin-2 levels and (A) age, (B) BMI, (C) VO_2peak_, and (D) glucose levels. Values are mean and 95% CI. Line represents fitted linear regression and shaded areas represent observed 95% CI. In Figure 1A, shaded area represent predicted 95% CI.

The relationship between LCN2 and age was best represented by linear regression (Table 2). Every 1 yr increase in age was associated with 0.26 mg/L increase in LCN2 levels (95% CI [0.07, 0.45], *p* = .007) (Figure 1A). LCN2 reference values for each age group are presented in Table 3.

**Table 2:** Model fit metrics for relationship between LCN2 and age.

	Deviance	*p* value	AIC	BIC
**Linear regression**	1076.3	Ref	1080.3	10 860
**Cubic splines with 1 knots**	1076.3	0.881	1082.3	1090.8
**Cubic splines with 2 knots**	1074.4	0.399	1082.4	1093.7
**Cubic splines with 3 knots**	1074.0	0.532	1084.0	1098.1
**Cubic splines with 4 knots**	1074.0	0.697	1086.0	1102.9

Abbreviations: AIC: akaike information criteria; BIC: Bayesian Information Criteria.

*p* value is from likelihood ratio test comparing deviance of each model to the linear regression.

**Table 3:** Lipocalin-2 reference values based on age.

Age group (yr)	Female (%)	*n*	LCN2 (mg/L)
**20-29**	44.44	9	62.6 (25.4, 99.8)
**30-39**	51.85	27	65.2 (28.2, 102.2)
**40-49**	50.00	14	67.8 (30.9, 104.7)
**50-59**	45.00	20	70.4 (33.6, 107.2)
**60-69**	68.18	22	73.0 (36.1, 109.9)
**70-79**	76.00	25	85.6 (8.6, 112.6)
**80 and above**	57.14	7	78.0 (40.7, 115.2)

Data presented as mean (reference range).

There was no difference for LCN2 levels between females and males (beta; −0.42, [−7.47, 6.52], *p* = .904). However for every 1 kg/m^2^ increase in BMI, there was a 0.88 mg/L increase in LCN2 levels ([0.10, 1.66], *p* = .027, Figure 1B) and for every 1 mL/kg/min increase in VO_2peak_, there was a 0.38 mg/L decrease in LCN2 levels ([−0.63, −0.13], *p* = .003, Figure 1C). No significant relationship was found between LCN2 and glucose levels (all *p* = .151).

When adjusted for BMI, the relationship between age and LCN2 was attenuated slightly (beta = 0.21 [−0.00, 0.42], *p* = .055), while the relationship between BMI and LCN2 was attenuated (coef 0.50, [−0.36, 1.36], *p* = .253). Because there was a high correlation between VO_2peak_ and age (Pearson’s *r* = −0.835), adjustment for VO_2peak_ was not performed.

**Figure 2:**
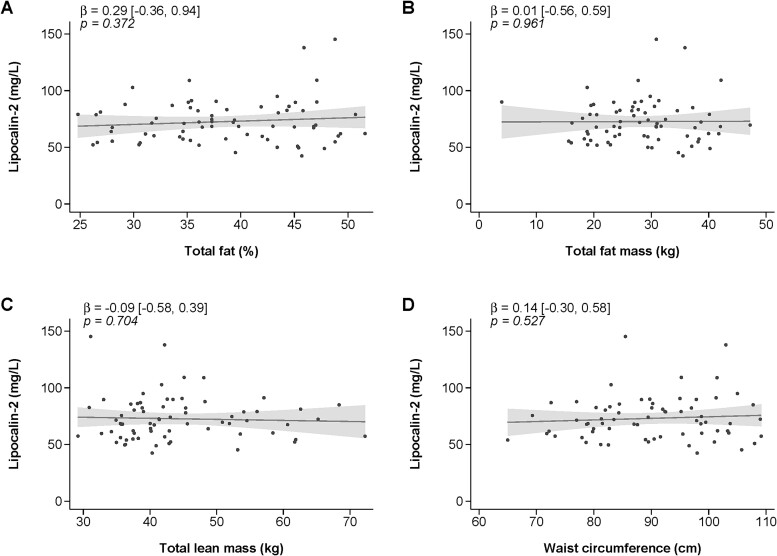
Linear regression of the relationship between lipocalin-2 levels and (A) total body fat %, (B) total body fat mass, (C) total body lean mass, and (D) waist circumference. Values are mean and 95% CI. *n* = 70 (males *n* = 20 [29%], females *n* = 50 [71%]). Line represents fitted linear regression and shaded areas represent observed 95% confidence interval.

Subgroup analysis of participants with available DXA measurements and waist circumference showed no significant relationships between LCN2 levels and body composition outcomes (total body fat % *p* = .372 Figure 2A; total body fat mass *p* = .961 Figure 2B; total body lean mass *p* = .704 Figure 2C; waist circumference *p* = .527 Figure 2D).

## Discussion

We report that (1) increased age is linearly associated with higher circulating LCN2 across the adult lifespan and (2) higher circulating LCN2 levels are associated with higher BMI and lower VO_2peak_. We provided potential reference ranges for every decade between the ages of 20 and 87 yr. Future studies should explore whether interventions to reduce LCN2 can prevent age-related diseases, such as cardio-metabolic disease, osteoporosis, and sarcopenia, and whether LCN2 can be used as a biomarker for chronic disease.

The aging population presents significant challenges to society due to an increased prevalence of chronic disease with age. Projected trends demonstrate that average lifespan will only continue to increase in the coming decades.^1^ As such, there is a need to investigate novel risk factors and potential treatment targets such as LCN2 for prevalent diseases in this population like cardio-metabolic diseases. LCN2, a bone and adipose-derived hormone, is associated with increased cardio-metabolic disease risk,^7^ however, differences across the lifespan have not been well characterized.

We reported that aging is related to higher circulating LCN2 levels across the adult lifespan in males and females. Clinically, the linear association between LCN2 and lifespan (age) may strengthen its utility for risk stratification, as it could be indicative of deterioration in cardio-metabolic processes as we age. Previous research has indicated that an acute increase in LCN2 may be the result of a protective mechanism in obesity-related metabolic dysfunction to suppress appetite and protect β-cell function in the early stages of disease.^24^ It is possible that transient increases in circulating LCN2 may have a protective effect, whereas chronic elevation may lead to pathological states. This may be similar to the phenomenon whereby acute hyperinsulinemia is an attempt to combat impaired glucose regulation in the early stages of insulin resistance development, however, chronic elevation becomes maladaptive and may promote pathological cardio-metabolic states.^25^ As aging is known to cause deterioration of physiological processes resulting in metabolic dysfunction, a similar protective mechanism may explain the elevated LCN2 levels in older adults. Future studies are needed to confirm this. This relationship aligns with previous studies indicating increased age is associated with higher circulating LCN2 levels, however, these studies did not examine the trends observed over the entire adult lifespan in both male and female sex,^15^^,^^16^ but were limited to females >50 yr and a small sample of highly fit, male ultra-athletes. In the current study, the relationship between LCN2 and age across the lifespan differed compared to other bone-derived hormones, osteoglycin and osteocalcin, that show a “U” shaped relationship.^13^^,^^14^ The different pattern may be due to the fact that osteoglycin and osteocalcin are predominantly released by bone while LCN2 is produced by a variety of cells from bone, adipose and other tissues. The clinical implications of the differences in the pattern across the lifespan is not yet clear. LCN2 may be another potential predictor for age-related health outcomes, future studies are needed to explore the age-related pattern in clinical populations.

We found sex had no effect on LCN2 levels across the lifespan in the current study. This finding is in contrast to a previous study that suggested circulating levels of LCN2 were higher in males compared to females, in lean (BMI < 23 kg/m^2^) and obese (BMI > 30 kg/m^2^) individuals (aged 33-72 yr).^8^ Previous research suggested that estrogen plays a role in adipose LCN2 expression that may be linked to sex differences.^8^ However, it is also suggested that tissue-specific LCN2 expression may be sex-specific in animal studies, especially for metabolism.^26^ White adipose LCN2 expression was associated with metabolic dysfunction in female, but not male mice, however, the mechanisms involved are unclear.^26^ In the current study, there was a significant difference between the mean age of males and females. However, we also report that both sexes demonstrated a similar pattern in circulating LCN2 levels across the adult lifespan. As such, further cross-sectional and longitudinal studies in humans with larger sample sizes are needed to fully explore sex differences in LCN2. Additionally, it will be important to explore the relationship between circulating LCN2 and tissue-specific LCN2 differences in future.

In the current study, higher circulating LCN2 levels were associated with lower VO_2peak_. This is consistent with observational reports that found a relationship between lower physical activity levels and higher LCN2 in community-dwelling older women.^18^ Additionally, training intervention studies have reported reductions in LCN2 in healthy sedentary young men (21-29 yr) who completed resistance or moderate continuous aerobic training for 8 wk and post-menopausal women (>45 yr) who completed resistance training for 15 wk,^27^^,^^28^ while a study in endurance athletes found LCN2 levels remained unchanged in response to training.^19^ Furthermore, a study found time-dependent increases in LCN2 with bed rest.^17^ As LCN2 is a mechanoresponsive gene, it is unclear whether mechanical loading during exercise contributes to lower LCN2 levels for people with higher VO_2peak_, other pathways stimulated by aerobic exercise, or a combination of both. Further intervention studies are needed to investigate whether LCN2 is a modifiable risk factor that can be targeted by chronic exercise training independent of weight changes in an aging population and the relationship to cardio-metabolic disease risk, as it is already well-established that physical inactivity is related to worsened outcomes for age-related disease and functional capacity.^29^ No relationship was observed between age and LCN2 when adjusting for VO_2peak_. Due to the high correlation between age and VO_2peak_, larger sample sizes would be needed to investigate this further.

LCN2 is an adipokine, with expression from adipose tissue increased in pathological states.^8^ We report that increases in LCN2 were significantly related to increases in BMI. Previous research supports a relationship between BMI and LCN2 across different populations including lean, obese, and overweight adults, individuals with diabetes, and community-dwelling older women.^8^^,^^18^^,^^24^ We previously reported the highest levels of LCN2 were related to increased BMI and associated with total body and appendicular fat mass in older women.^16^ It is important to note that in contrast to BMI, no significant relationship was observed between fat mass, lean mass or central adiposity (waist circumference) and LCN2 levels in the current study. It is possible that the lack of relationship between LCN2 with total body fat mass and lean mass is due to a smaller sample size (*n* = 70) and age range (45-87 yr) for these variables as these data were available for a subset of participants. Future studies should explore the relationship between LCN2 and body composition parameters further with both cross-sectional and longitudinal studies.

As previously described, there are some potential limitations in this study. The study design combined cohorts from separate studies with slightly different exclusion criteria and study design. All participants in the current study were free of overt cardiovascular and metabolic disease, however, it’s plausible that some participants had unidentified or subclinical conditions. Additionally, all samples were collected in the morning, in a fasted state. LCN2 levels measured can vary between studies due to differences in the analysis method and antibody used therefore, reference ranges may differ if other analysis methods are used. A strength of the current study is that all samples were analyzed in the same laboratory, using the same analysis techniques within the same time-period. As described previously, LCN2 is an established marker of kidney injury and as such, it is possible that the levels of LCN2 across the aging range is, at least in part, related to kidney function. However, it is unlikely that estimated glomerular filtration rate (eGFR) would be compromised in these individuals included in the study based on inclusion criteria. Due to the current study design, kidney function, as well as bone turnover markers, was not assessed. While this study provides important insights into the differences in LCN2 levels over the adult lifespan, longitudinal studies are needed to determine how chronic changes over the lifespan relate to cardio-metabolic risk and disease.

In conclusion, LCN2 increased across the lifespan in adults free of major disease. LCN2 also increased with higher BMI and decreased with higher aerobic fitness, independent of sex. Future studies should elucidate the clinical implications of changes to LCN2 across different clinical populations and explore whether interventions to reduce LCN2 can prevent age-related disease risk or severity.

## Data Availability

Datasets generated during the current study are not publicly available but are available from the corresponding author on reasonable request. This material will be available on request and approval of Victoria University Human Ethics Committee.

## Clinical trial registration

Australia New Zealand Clinical Trials Registry ACTRN12618001756213, ACTRN12622000337774, and ACTRN12613000706774.
